# Dynamic Mixed Data Analysis and Visualization

**DOI:** 10.3390/e24101399

**Published:** 2022-10-01

**Authors:** Aurea Grané, Giancarlo Manzi, Silvia Salini

**Affiliations:** 1 Department of Statistics, University Carlos III of Madrid, 28903 Getafe, Spain; 2 Department of Economics, Management and Quantitative Methods and Data Science Research Center, University of Milan, 20122 Milano, Italy

**Keywords:** mixed data, robustness, outliers, time series, data visualization

## Abstract

One of the consequences of the big data revolution is that data are more heterogeneous than ever. A new challenge appears when mixed-type data sets evolve over time and we are interested in the comparison among individuals. In this work, we propose a new protocol that integrates robust distances and visualization techniques for dynamic mixed data. In particular, given a time t∈T={1,2,…,N}, we start by measuring the proximity of *n* individuals in heterogeneous data by means of a robustified version of Gower’s metric (proposed by the authors in a previous work) yielding to a collection of distance matrices {D(t),∀t∈T}. To monitor the evolution of distances and outlier detection over time, we propose several graphical tools: First, we track the evolution of pairwise distances via line graphs; second, a dynamic box plot is obtained to identify individuals which showed minimum or maximum disparities; third, to visualize individuals that are systematically far from the others and detect potential outliers, we use the proximity plots, which are line graphs based on a proximity function computed on {D(t),∀t∈T}; fourth, the evolution of the inter-distances between individuals is analyzed via dynamic multiple multidimensional scaling maps. These visualization tools were implemented in the Shinny application in R, and the methodology is illustrated on a real data set related to COVID-19 healthcare, policy and restriction measures about the 2020–2021 COVID-19 pandemic across EU Member States.

## 1. Introduction

Statistical comparison among objects (individuals, groups, regions, countries, etc.) is a wide area involving several aspects. At the very basis of any comparison is the concept of similarity (or dissimilarity) among these objects from which other analyses can be performed. The huge availability of data of different type (continuous, discrete, categorical, binary, etc.), which is a consequence of the big data revolution, pushes researchers to find methods for measuring such object similarities such that they are not unduly affected by outliers and anomalies in the data. An even more challenging task is when one has to deal with mixed data when the definition of outliers depends on the nature of the different data types. Moreover, when data presents in the form of time series, an optimal object comparison might be very hard to achieve.

A recent example is the huge amount of information produced due to the COVID-19 pandemic, for which not only are daily epidemiological data available, but so are people’s mobility trends, stringency measure variables in different European regions, etc. If we are interested in finding which EU state member looks more similar (or more distant) in the management of the pandemic, or even whether there is a country systematically different from the others, it seems logical to use all the available statistical information to derive statistical proximities among countries. Data sets of that type and such questions were the motivation of this work. Our main interest is in developing a protocol to compare the similarities (dissimilarities) among a set of heterogeneous data over time and produce graphical tools to monitor their evolution.

In case of mixed data, the choice of the distance metric to be used for clustering or dimensional reduction problems has been widely discussed in recent years. Foss et al. [1] give a thorough review of distance-based clustering methods for mixed data. They essentially divide these methods into data transformation methods, hybrid distance methods involving distance functions that can accommodate both interval and categorical scale variables and statistical mixture models. Bar-Hille et al. [2], proposed a relevant component analysis algorithm, which is a simple and efficient algorithm for learning a Mahalanobis metric, aiming at changing the variable space using a global linear transformation which assigns large weights to “relevant dimensions” and low weights to “irrelevant dimensions” through the use of *chunklets*, i.e., small subsets of points that are known to belong to the same unknown class, instead of the variance-covariance matrix in the Mahalanobis distance. Jian et al. [3] used a metric-based algorithm for mixed data representation. This representation learns the couplings between categorical and continuous variables at the variable level, and the separation between objects at the object level. Wang et al. [4] sought for robust distance metric learning via Bayesian inference. In the context of multidimensional scaling (MDS), Grané and Romera [5] compared multiple MDS configurations for profiling objects using a robust joint metric combining different distance matrices via related metric scaling [6,7].

Grané et al. [8] recently proposed a novel algorithm for the robust analysis and clustering of mixed-type data using a hierarchical approach based on the combination of a proximity function and the forward search algorithm [9]. Their proposal was successful in discarding redundant information from a given set of variables, unlike classical Gower’s distance [10], although the metric’s computation can be computationally expensive. For this purpose, Grané et al. [11] proposed a protocol based on the combination of this algorithm and robust clustering, by means of a robustified version of Gower’s metric.

The main task of this paper is to extend Grané et al.,’s protocol [11] for similarity (dissimilarity) comparisons over time. In particular, given a time t∈T={1,2,…,N} and a set of *n* individuals with heterogeneous measurements, we performed the comparisons over time using robust distance matrices for mixed data evaluated at each time *t*, and developed tools for smart visualization to monitor the evolution of inter-distances over time. We used mixed data from 25 out of 27 EU countries related to the spread of the COVID-19 pandemic from January 2021 to March 2022. Data include health variables, people’s mobility variables and stringency measure variables (school closures, etc.).

The paper is organized as follows. In Section 2, we present an overview of the underlying methods and propose an algorithm for monitoring comparison over time. In Section 3, we show the application of the algorithm and the protocol to the COVID-19 data set produced by ourselves, and we conclude in Section 4. For the comparison visualization, we developed three Shiny applications in R which can be retrieved from https://github.com/giancaman/Manuscript-Dynamic-mixed-data-analysis-and-visualization-apps. The data sets and the instructions for running the apps are also contained in this GitHub repository, along with the Matlab and R code.

## 2. Materials and Methods

Let T={1,2,…,N} be a set of times. For each time t∈T, let $\left\{z_{i}\left(t\right),i=1,\ldots ,n\right\}$ be a set of vectors of $R^{p}$ of mixed-type data, which correspond to the observations of *p* variables of different natures on a sample of *n* individuals.

We are interested in measuring the statistical proximity among *n* individuals at time *t*. For that purpose, we select an adequate distance measure δ and consider $D\left(t\right)={\left(\delta^{2}\left(z_{i}\left(t\right),z_{j}\left(t\right)\right)\right)}_{1\leq i,j\leq n}$ a matrix of squared pairwise distances at time *t*.

Following Cuadras and Fortiana [12], we define the geometric variability of D(t) as

(1) $$V_{D(t)}=\frac{1}{2n^{2}}1^{\prime }D\left(t\right)1=\frac{1}{2n^{2}}\sum_{i,j=1}^{n}\delta^{2}\left(z_{i}\left(t\right),z_{j}\left(t\right)\right),$$

where 1 is a column vector of ones of size *n*. The concept of geometric variability was introduced in [12] as a generalization of the total variability of a random vector, and it is related to Rao’s quadratic entropy [13], in the context of diversity measures.

### 2.1. A Robust Metric to Track Proximity over Time

Gower’s distance [10] is perhaps the most popular distance for mixed data. It was defined for static mixed variables as the Pythagorean sum of three distance measures for quantitative, binary and multi-state categorical variables, $D_{1}=\left(\delta_{1}^{2}\left(z_{i},z_{j}\right)\right),D_{2}=\left(\delta_{2}^{2}\left(z_{i},z_{j}\right)\right)$ and $D_{3}=\left(\delta_{3}^{2}\left(z_{i},z_{j}\right)\right)$, where $\delta_{1}$ is the range-normalized city-block distance, $\delta_{2}$ distance is associated with Jaccard’s similarity coefficient and $\delta_{3}$ is the Hamming distance.

However, this well-known distance presents two main drawbacks: First, it does not take into account the correlations between quantitative variables; therefore, the distance always increases when new quantitative variables are included in the data set, even if they do not contain new statistically relevant information. Second, it is a not robust metric; thus, it can lead to non-stable MDS configurations [5,8]. Finally, outliers cannot be easily located in MDS configurations obtained with Gower’s metric [5,11].

To circumvent these problems, Grané et al. [11] proposed a robustification of Gower’s distance, by taking $\delta_{1}$ as a robust Mahalanobis distance, instead of range-normalized city-block distance, and they left $\delta_{2}$ and $\delta_{3}$ unchanged.

In order to track proximity over time, we propose to extend the previous idea to each time t∈T and to different data sources. Thus, for each t∈T we define the following joint robust metric:

(2) $$D\left(t\right)=\sum_{k=1}^{m}D_{k}\left(t\right)=\sum_{k=1}^{m}\left(\delta_{k}^{2}\left(z_{i}\left(t\right),z_{j}\left(t\right)\right)\right),$$

where *m* is the number of data sources to be combined, such as variable type, source of information or both, and with the additional requirement that $V_{D_{k}\left(t\right)}=1$ for k=1,…,m. A robust Mahalanobis distance is computed for quantitative variables; distance associated with Jaccard’s similarity coefficient and Hamming distance are considered for binary and multi-state categorical variables, respectively. Regarding the additional requirement, equal geometric variability can always be assumed to hold, since multiplying a matrix of squared distances by an appropriate constant amounts to a change in measurement unit.

### 2.2. Tracking a Source of Variability over Time

Formula (1) defines a collection of geometric variabilities over time, and when applied to each matrix $D_{k}\left(t\right)$, for k=1,…,m, it also allows one to track the variability of the *k*th source of data over time, that is, $V_{D_{k}\left(t\right)}$, for t=1,…,N.

We propose to visualize $V_{D_{k}\left(t\right)}$, for k=1,…,m, t=1,…,N, in a line graph in order to analyze the influence of each source in the global variability over time.

In the application below, this concept is used to analyze the variability of health data, stringency data and mobility data and how they relate to each other over time.

### 2.3. Distance Visualization over Time

Formula (2) defines a collection of distance matrices over time {D(1),…,D(N)} that yield the possibility to track the pairwise distances of each individual to the others over time. We propose to visualize the evolution of these pairwise distances in a line graph.

For example, in the application below, we fixed one country and studied the evolution of its pairwise distances to a set of countries over time. This type of analysis is included in the R Shinny application.

A very interesting analysis that is derived from the study of matrices {D(1),…,D(N)} is to search for the most similar and most dissimilar individuals in the considered period. For this purpose, for each *t*, we compute the minimum and maximum pairwise distances, plot them in a line graph and identify those individuals with minimum and maximum distances. Other extensions of this kind of analysis would be to plot the first and third quartiles of the pairwise distances, mimicking a dynamic box plot.

Another interesting analysis that we propose is to plot the proximity of each individual to the others over time in a line graph. This kind of analysis turned out to be very useful in order to detect which individuals are systematically far from the others and to discover potential outliers. Following Cuadras et al. [14], we define the dynamic proximity function of a given individual with observed variables $z_{0}\left(t\right)\in R^{p}$ to $\left\{z_{i}\left(t\right),i=1,\ldots ,n\right\}$ as

(3) $$\phi \left(z_{0}\left(t\right)\right)=\frac{1}{n}\sum_{i=1}^{n}\delta^{2}\left(z_{0}\left(t\right),z_{i}\left(t\right)\right)-V_{D(t)},\;t\in T,$$

where $V_{D(t)}$ is the geometric variability of D(t). This function takes higher values as $z_{0}\left(t\right)$ departs from the set $\left\{z_{i}\left(t\right),i=1,\ldots ,n\right\}$, but taking into account the geometric variability of the set. Thus, large values of the ϕ-function indicate a possible outlier.

### 2.4. Dynamic Multiple MDS Maps

MDS is the usual multivariate technique to visualize pairwise distances between individuals. In Grané et al. [8], multiple MDS maps were defined to visualize clusterings and outliers in MDS configurations. Now, we propose to use the collection of distance matrices {D(1),…,D(N)} defined in Formula (2) to construct a dynamic plot by concatenating the corresponding multiple MDS maps associated with each D(k).

**Figure 1:**
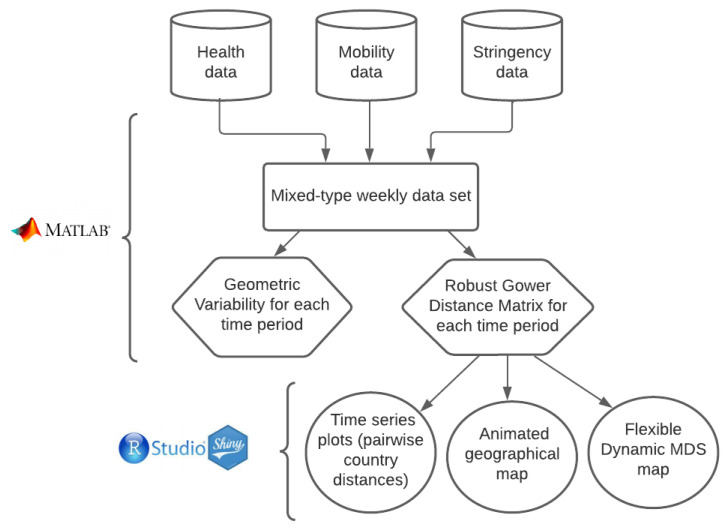
Flow chart of the process.

To summarize the procedure that we propose, in Figure 1 we include a flow chart describing the process.

## 3. Results

### 3.1. Data Set Construction

To apply our method, we created a time series data set with variables related to the spread and policies adopted by EU member states for the COVID-19 pandemic from 1 February 2021, to 14 March 2022, comprising 59 weeks. We chose 15 variables divided in three groups: (i) six healthcare variables related to the spread of COVID-19 (5 discrete quantitative variables: COVID-19 cumulative new cases, deaths, tests, people hospitalized and people in intensive care units; 1 percentage variable: people fully vaccinated per hundred); (ii) stringency variables related to governmental decisions to tackle the pandemic’s spread (three index variables with values ranging from 1 to 100: government response index, stringency index and containment health index); (iii) mobility variables related to people’s movements during the pandemic (six percentage change variables from a reference baseline date fixed just before the start of the pandemic: mobility from and to retail and recreation spots, mobility to and from groceries and pharmacies, parks, transit railway stations, workplaces and residential movements). Variables in group (i) were taken from data repositories for COVID-19 variables [15,16]. Variables in group (ii) were retrieved from the database described in [17] about monitoring the global COVID-19 government response, whereas variables in group (iii) were formed using Google mobility trend data (www.google.com/covid19/mobility, (accessed on date 1 June 2022). Data on mobility for Luxembourg and Cyprus were not available at the time we collected the data, so we dropped these two countries from the analysis. After transforming the healthcare variables in the country to per 1000 or per 100 inhabitants, we finally picked the values of the first day of each week for each country, which were the data inputs for our analysis.

### 3.2. Visualizing Time Series Data

In Grané et al. [11], we divided the epidemiological time series into three periods: the first one corresponds to the first wave of the pandemic with western EU countries more affected than eastern EU countries (from the end of February 2020 to mid-June 2020), the second one to the summer of 2020 when the pandemic was less strong almost in every EU country, and the final one corresponds to the second pandemic wave which affected almost all the EU countries (from mid-September 2020 to end of November 2020). The three periods are referred to as Wave 1, Wave 2 and Wave 3 in the following. In that paper, even if original data were time series data, our analysis was aimed at exploring the overall characteristics of the different waves in the EU countries using visual tools. For this reason, we aggregated our data according to: type of variable, wave, country and aggregating statistics. The statistics considered for aggregation were mean, median and maximum for quantitative variables, and the mode for qualitative variables (including the only binary variable in the data set, i.e., the country’s health system type).

**Figure 2:**
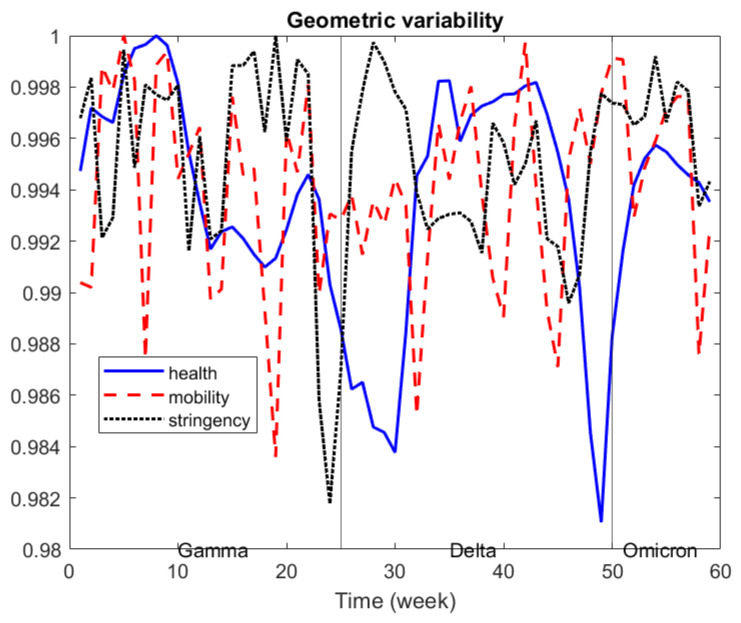
Geometric variability over time by source of data.

In Figure 2, we depict the evolution of the geometric variability over time, taking into account the three sources of information, and we re-scaled it to 0–1 for better comparison. Health data variability show a relative decrease by the end of each Covid-variant period, which seems to indicate that differences across countries tended to decrease before a new Covid-variant appears. Similar behavior can be seen for stringency data, whereas mobility data show a very irregular pattern.

**Figure 3:**
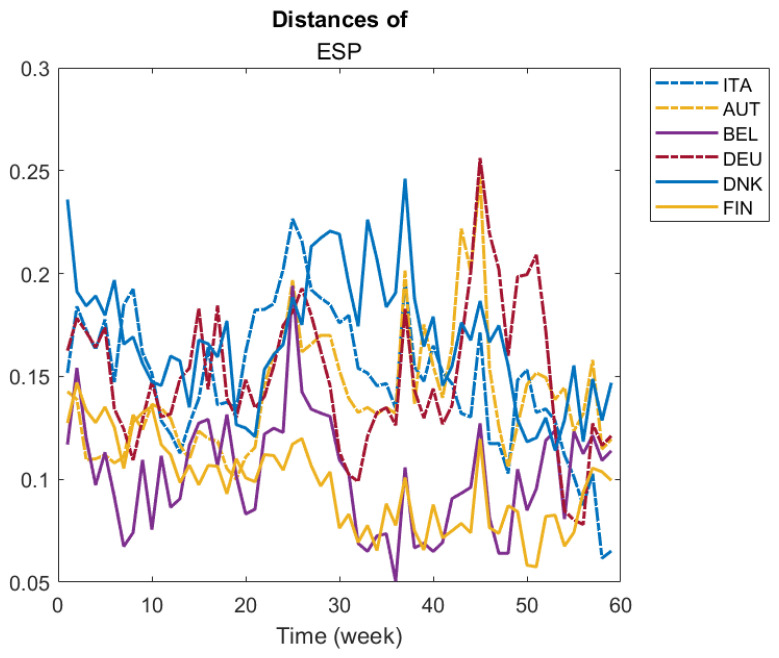
Evolution of pairwise distances of a given country to a set of countries over time.

In Figure 3, we illustrate the evolution of pairwise distances of a given country to a group of selected countries. We selected four countries, Italy, Spain, Germany and Denmark, to illustrate their pairwise distance evolution compared to the following set of countries: Italy, Spain, Austria, Belgium, Germany, Denmark and Finland. For example, looking at ITA panel, we see that from week 30th onwards, Denmark was the most different country from Italy. On the other hand, Belgium and Austria seem to have been the most similar to Italy throughout several weeks of the period. Additionally, Belgium seems to be very similar to Germany during most of the period (see DEU panel), and Finland was the most similar to Denmark in most of the period (see DNK panel).

**Figure 4:**
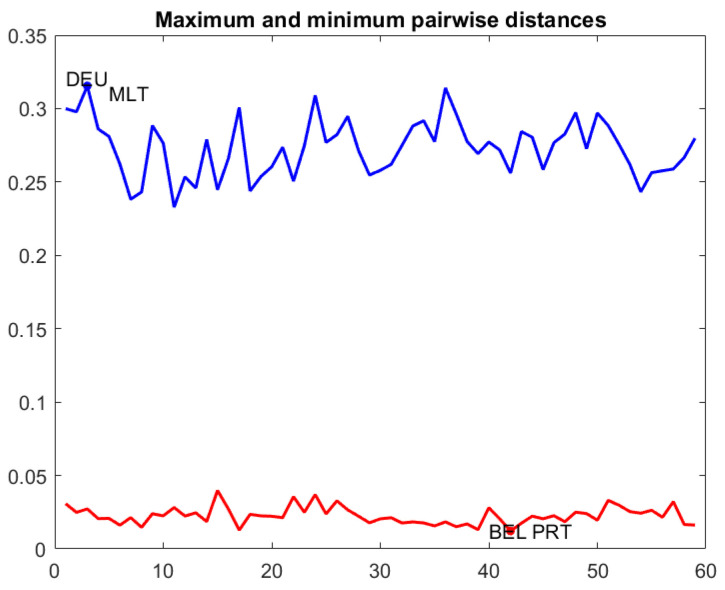
Maximum and minimum pairwise distances over time and dynamic box plot for pairwise distances.

Figure 4 contains a line graph with the maximum and minimum pairwise distances throughout the period, and a dynamic box plot for pairwise distances. The first plot is useful to detect the most similar countries at a given time *t* and the most distant ones. For example, the farthest countries were Germany and Malta at the 3rd week of the period of study, whereas the nearest ones were Belgium and Portugal at 42nd week. The dynamic box plot helps to delimit the range of the 50% central pairwise distances.

**Figure 5:**
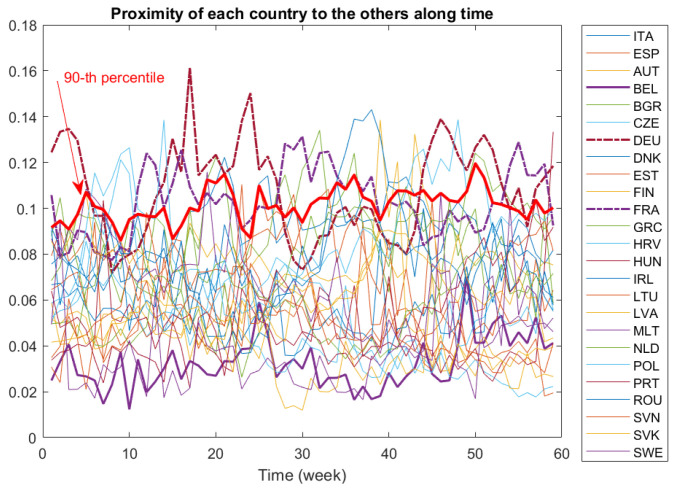
Proximity of each country to the others over time.

Comparisons among countries may be difficult by looking only at pairwise distances. Indeed, a dynamic proximity function is a better tool to search for the closest or the farthest country to the others along all the period. In Figure 5, we depict the dynamic proximity function defined in (3) of each country to the others. Recall that the greater the value of the this function, the farther is this country from the others. This may be the case for Germany (DEU) and France (FRA), whose ϕ-function values are greater than the 90th percentile during most of the period. Conversely, Belgium (BEL) is the country most similar to the others, at least before the 50th week of study.

The distance matrices and graphs in Figure 2, Figure 3, Figure 4 and Figure 5 were produced using Matlab.

### 3.3. R Shiny Applications to Dynamically Monitor Countries’ Distances over Time

To facilitate understanding of our method, we have further improved the above visualization tools by using all the distance matrices for each week as an input for three *shiny applications* developed in R. The first application is about showing time series of distances between one reference country and other chosen countries; in the second one, the reference country is compared to all the other countries in a map where different colors are assigned to countries, the color grading being proportional to the distances with respect to the reference country; the third one is a scatter plot showing the countries with coordinates given by the first two MDS axes and with circles representing countries with sizes proportional to either new COVID-19 cases or the number of vaccinated people. The last two shiny applications allow the user to dynamically monitor the comparison along the entire 59-week period using a slider object in the application. All the three applications start by asking the user to choose the location where the input data sets have been stored.

#### 3.3.1. Application 1: Time Series

**Figure 6:**
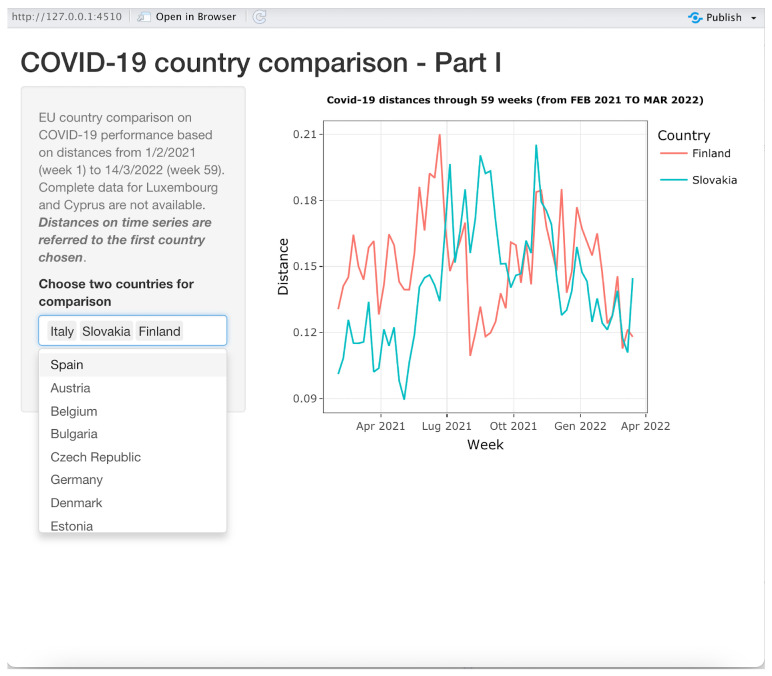
Application I: Distance time series display.

This application is illustrated in Figure 6. It produces time series graphs similar to those shown in Figure 3.

The first chosen country in the drop-down list control on the bottom-left side is the reference country. In the example shown in Figure 6, the distances between Italy and Slovakia and Finland over all 59 weeks are shown. By canceling “Italy” from the select list control, the application will automatically display the time series of the distance between Slovakia and Finland. The user can choose up to 24 countries to be compared to the reference country on the same time series plot. The currently displayed plot can be saved, printed or zoomed in on using functions made available when the pointer is moved on the right top corner of the plot.

#### 3.3.2. Application 2: Using a Map for Comparison

**Figure 7:**
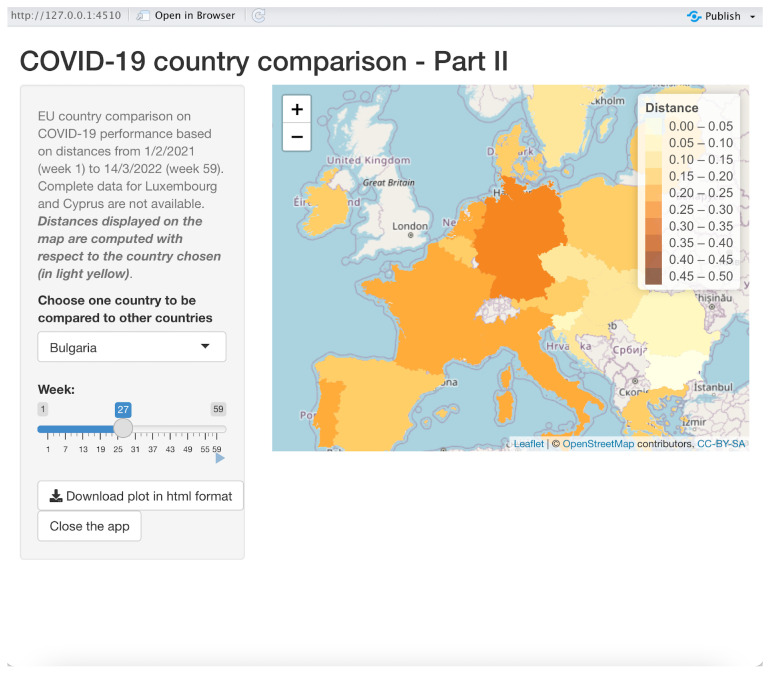
App II: Distance map.

This application is illustrated in Figure 7. It produces animated maps to dynamically compare a reference country to all the other 24 countries.

Once the user has chosen the reference country in the drop-down list control on the left-hand side of the window, a map is displayed, showing the distances of the other countries with respect to the reference country, which will appear in light yellow. A different color is used for different distances; a legend explains the magnitudes of the distances. The user can choose the week(s) with the related distances by using the slider “Week” on the left-hand side of the window. One can see the dynamics of the distance distributions of the countries with respect to the reference country by using the tiny start button right in the bottom right corner of the slider, producing an animation with a new map on each different week. Once the user clicks on this button, a stop button appears, which can be used to stop the animation. The slider can be used to choose a particular week by dragging and dropping the slider’s thumb, i.e., the position indicator that can be moved along the slider’s track, showing the current slider’s value (the number of the week in our case). One can change the reference country, producing an immediate refresh of the map, which will have the new reference country colored in light yellow. This can be done “on the fly” even when the animation is in progress. A download button is available to download the current map in HTML google maps format, and a zoom tool is displayed on the top right-hand corner of the map.

In the example showed in Figure 7, Bulgaria is the reference country in week 27: one can immediately be aware that Germany is the most distant country from Bulgaria in that week, whereas Romania has the lowest distance from Bulgaria.

#### 3.3.3. Application 3: Displaying Principal Coordinates for Comparison

**Figure 8:**
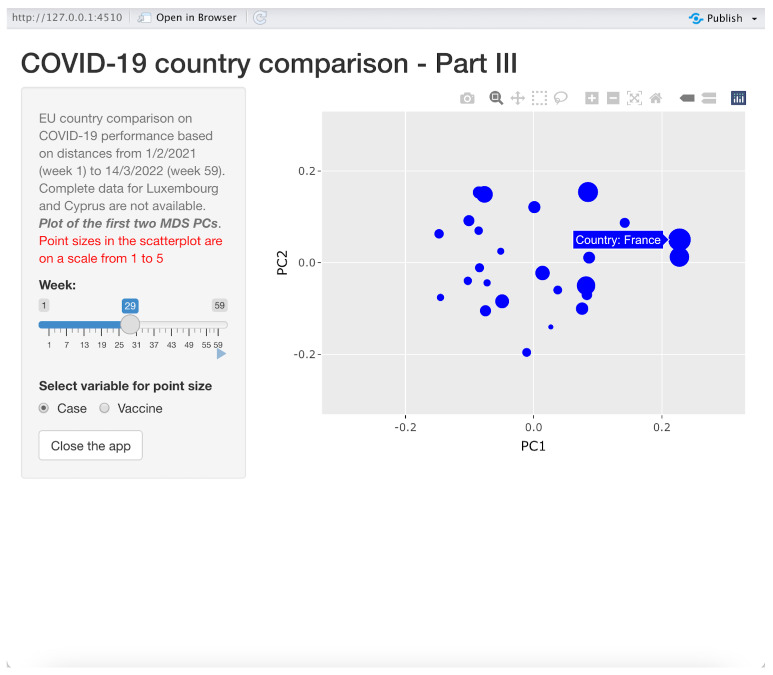
App III: PCs from MDS with case/vaccines magnitude.

This application is illustrated in Figure 8 and produces animated scatter plots to dynamically represent all the considered countries in a space spanned by the first two principal coordinates (MDS PCs).

As the example in Figure 8 clearly shows, countries are represented (in a given week chosen using a slider similar to the one in application 2) in the two-dimensional space formed by the first two MDS PCs. The sizes of the circles are proportional either to the number of COVID-19 cumulative cases or to the number of cumulative vaccinations in the chosen week. By moving the pointer on the dots, the name of the country appears. In the example, France is the selected country. Again, the current scatter plot can be downloaded, printed and zoomed in on, and an animation can be seen using the slider. The dot sizes are re-scaled in a range between 1 and 5. One can choose the proportion variable (number of cases or number of vaccinations) by switching between “Case” and “Vaccine” in the option button on the bottom left-hand side of the window.

## 4. Discussion

In many statistical fields, but above all in modern machine learning, as far as the availability of data sources increases, methods must be flexible enough to be applied to any sort of data, from numerical to categorical, taking in due account the mixed nature of the data [18]. One of the main problems when dealing with mixed data is to maintain adequate robustness in the estimators or in the data representation. When one wants to monitor a certain phenomenon over time, these problems are even more striking due to the increased presence of extreme values that are not completely comparable from one moment in time to another.

Building on the work of Grané et al. [11], in this paper we presented several contributions to solving the problem of a robust representation of data points in a temporal sequence, starting from using a robust distance metric and studying the evolution of the dissimilarity over time.

The proposed procedure can answer questions such as: Does the dissimilarity between statistical units increase over time? What are the periods where the differences between statistical units are minimal or maximal? What are the statistical units that are more divergent?

Focusing more on the particular application presented in this paper, we can answer some important questions: During the COVID-19 pandemic, which countries behaved more differently from others? Over the course of the various weeks and variants, did the overall situation move toward being more homogeneous, or did the differences increase? Were the overall healthcare situation and the various interventions applied by different countries in Europe more or less similar? Which countries stood out, and during which periods?

An important part of our contribution concerns visualization. Matlab functions were implemented to calculate distance matrices for the various time periods, and three R shiny applications were implemented allowing for flexible user-friendly visualizations (time series, geographic maps, dimensional maps), the choice of countries to compare and the choice of variables to integrate. Regarding the time complexity of the robust representation of the data points, which is another important feature of our work if one thinks about the dynamic of the distance matrices’ construction (i.e., the computation of 59 weekly distance matrices for 25 countries), the execution time for the Matlab code was 7.38 s without graphs, and 10.68 s with graphs (more than 30 plots produced). This code was executed on a Toshiba laptop; processor: Intel(R) Core(TM) i7-5500U CPU @ 2.40 GHz; RAM: 16.0 GB; 64-bit Windows operating system, x64-based processor.

One of the uses of this contribution is that it could be applied to all contexts in which diversity over time and its evolution need to be measured. It starts with distance matrices, which can be obtained in a variety of ways, from classical methods to more complex and robust methods for mixed data. It is a kind of three-way analysis. The number of observations, the number of variable and the number of periods do not matter.

There are many application areas where the method can be generalized and could have a good impact. For example, ref. [19] measures the diversity of higher education institutions. The work in [20] classifies trajectories of Italian regions by their employment dynamics.

In future, we would like to focus on the problem of outlier identification in distance matrices instead of variables, and the mining of outliers in distance matrices over time. We want also to implement distance measures that are different from the robust Gower distance and to study the sensitivity of the results with respect to the selected distance type. Another future direction that might arise from this contribution is the attempt to create tree-way MDS-maps, where the PCs and the factor loadings are comparable over time.

Finally, it is our intention to harmonize all the functions produced for this paper and produce a complete R package that, starting from the mixed-type raw data, implements user-selected distances and allows all possible visualizations to be obtained interactively.

## Data Availability

Data sets and code are available on the GitHub repository https://github.com/giancaman/Manuscript-Dynamic-mixed-data-analysis-and-visualization-apps. In the future, the Matlab part might be included in the FSDA Matlab toolbox.
